# Chimpanzees (*Pan troglodytes*) Flexibly Use Introduced Species for Nesting and Bark Feeding in a Human-Dominated Habitat

**DOI:** 10.1007/s10764-016-9916-y

**Published:** 2016-09-16

**Authors:** Maureen S. McCarthy, Jack D. Lester, Craig B. Stanford

**Affiliations:** 1 Department of Biological Sciences, Dana and Dornsife College of Letters, Arts, and Sciences, University of Southern California, Los Angeles, CA 90089-0371 USA; 2 Department of Primatology, Max Planck Institute for Evolutionary Anthropology, 04103 Leipzig, Germany

**Keywords:** Bark feeding, Chimpanzee, Eucalyptus nesting, Fragmented habitat, *Pan troglodytes*

## Abstract

As habitat loss and fragmentation place growing pressure on endangered nonhuman primate populations, researchers find increasing evidence for novel responses in behavior. In western Uganda between the Budongo and Bugoma Forests, chimpanzees (*Pan troglodytes schweinfurthii*) inhabit a mosaic landscape comprising forest fragments, human settlements, and agricultural land. We recorded nests and feeding evidence of unhabituated chimpanzees in this region over a 12-mo period. We found extensive evidence of nesting in introduced tree species, including eucalyptus (*Eucalyptus grandis*), guava (*Psidium guajava*), cocoa (*Theobroma cacao*), and Caribbean pine (*Pinus caribaea*). In addition, we found instances of ground nesting, nest reuse, and composite nests constructed from branches of multiple trees. This evidence may indicate a lack of suitable nesting trees or attempts by chimpanzees to nest in areas of riparian forest that allow them to avoid human detection. We also found new evidence for eucalyptus bark feeding by chimpanzees. Such evidence suggests chimpanzees respond flexibly to mitigate anthropogenic pressures in human-dominated landscapes. The limits of such flexibility remain unknown. Further research is needed to examine systematically the factors influencing the use of such resources and to understand better the extent to which chimpanzees can persist while relying on them.

## Introduction

Anthropogenic habitat changes can impact primate behavior in a number of ways. For example, great apes may alter their activity budgets and travel patterns in human-disturbed environments (Ancrenaz *et al*. 2014; Campbell-Smith *et al*. 2011; Cibot *et al*. 2015; Hockings *et al*. 2012; Krief *et al*. 2014). They may also respond to habitat changes by incorporating new species into their diets. For example, orangutans feed on oil palm trees in plantations in Borneo (Ancrenaz *et al*. 2015). Chimpanzees at numerous research sites under varying levels of human impact also consume cultivars (Bessa *et al*. 2015; Hockings and McLennan 2012; Hockings *et al*. 2009; Krief *et al*. 2014; McLennan 2013; Reynolds 2005). Additionally, mountain gorillas feed on eucalyptus bark in tree plantations (Rothman *et al*. 2006). Such behavioral responses provide insights into great ape cognition and evolution, and can provide valuable data to guide conservation efforts for remaining populations (Hockings *et al*. 2015).

Chimpanzees and other great apes may also alter their nesting patterns in response to human disturbance, e.g., by varying nest height. For example, a higher prevalence of ground nesting has been reported where human density was lower, suggesting chimpanzees may nest more freely on the ground when human pressure does not inhibit this behavior (Hicks 2010; Last and Muh 2013). In contrast, another study reported more ground nesting at times when human pressure was higher (Tagg *et al*. 2013). The authors suggested ground nesting in this context may be enabled by the chimpanzees’ increased use of swamp forest, a habitat rarely entered by humans, thereby facilitating ground nesting and lessening the likelihood of human detection. Such pressure could hypothetically drive low nest heights as well. Low nests and ground nests could also result from a limited availability of suitable nesting trees, however, leading individuals to nest in smaller, less sturdy trees or on the ground by necessity. Therefore, although night ground nesting has been reported at a number of research sites (Furuichi and Hashimoto 2000; Koops *et al*. 2012; Pruetz *et al*. 2008), variations in its frequency may sometimes be related to human disturbance for numerous reasons.

Other changes to nesting patterns may be expected to occur in human-impacted great ape habitats as well. As favored nest tree species are felled, other introduced species may replace them, either by necessity, due to the adoption of new nest tree preferences, or both. (In this article we use the term *introduced species* to refer broadly to exotic, nonnative species that are grown for food, timber, or fuel wood.) For example, Bornean orangutans, as well as chimpanzees in Guinea-Bissau, nest in oil palm trees (Ancrenaz *et al*. 2015; Sousa *et al*. 2011). Lower nest tree availability may also lead great apes to reuse nests, as low availability of fresh leaves may contribute to nest reuse (Fruth and Hohmann 1996; Wrangham 1992). A lower availability of tall, sturdy nest trees in logged habitat may also lead to the construction of composite nests composed of smaller, less sturdy trees and other vegetation. Few studies have documented great ape nesting patterns in human-disturbed habitat, however, and the use of introduced species for nesting has rarely been reported to date.

In a roughly 1200-km^2^ area in western Uganda between the Budongo and Bugoma Forests, chimpanzees (*Pan troglodytes schweinfurthii*) inhabit small forest fragments. Using noninvasively collected fecal samples for genetic analysis, a recent study identified a total chimpanzee population numbering 256 (95 % CI: 246–321) or 319 (288–357) chimpanzees, depending on the estimator used (McCarthy *et al*. 2015). Genotypes clustered in nonoverlapping polygons distributed throughout the study area, suggesting the presence of a minimum of nine distinct chimpanzee communities. These chimpanzees inhabit mostly the unprotected riparian forests in this area, but range through the agricultural matrix and consume cultivars from local homesteads and gardens (McLennan 2013), sometimes coming into conflict with their human neighbors (McLennan and Hill 2012).

These conflicts appear more common as human pressures increase (McLennan and Hill 2012). Human population density is estimated at 156.6 and 113.3 residents per km^2^ respectively in Hoima and Masindi Districts, the two governmental districts comprising this region (UBOS 2014). Uganda’s human population growth rate is 3.8 %, one of the highest globally (CIA 2013). Much of the human population relies on subsistence and commercial agriculture (FAO 2010), and 90 % of Ugandans rely on wood fuel as a main energy source (MWLE 2002). These growing pressures have led to a 37 % forest loss in Uganda from 1990 to 2010 (FAO 2010), with >130 km^2^ of forest loss in the region between Budongo and Bugoma in the past several decades (Twongyirwe *et al*. 2015). Plantation forests have replaced natural forests in some areas and play an integral role in modern forestry policy in Uganda (Turyahabwe and Banana 2008). However, the ability of plantation forests to reduce reliance on natural forests is questionable (Ainembabazi and Angelsen 2014).

Given the ongoing chimpanzee habitat loss in this region and the increasing prevalence of introduced species, it is critical to understand how Endangered chimpanzees respond to such habitat changes to better predict their likelihood of survival and to devise appropriate conservation strategies. The purpose of this study was to document nesting patterns among chimpanzees in this fragmented forest landscape to determine how nesting preferences may reflect responses to anthropogenic habitat changes, including the increasing prevalence of introduced species. We hypothesized that chimpanzee nesting patterns would reflect behavioral responses to habitat disturbance. In particular, we predicted that chimpanzees would nest in introduced species, reuse nests, and construct nests from multiple trees. Further, we predicted that chimpanzees would nest at low heights or on the ground. In addition, we opportunistically documented evidence of a novel feeding behavior by chimpanzees in this environment.

## Methods

### Study Area and Subjects

**Fig. 1:**
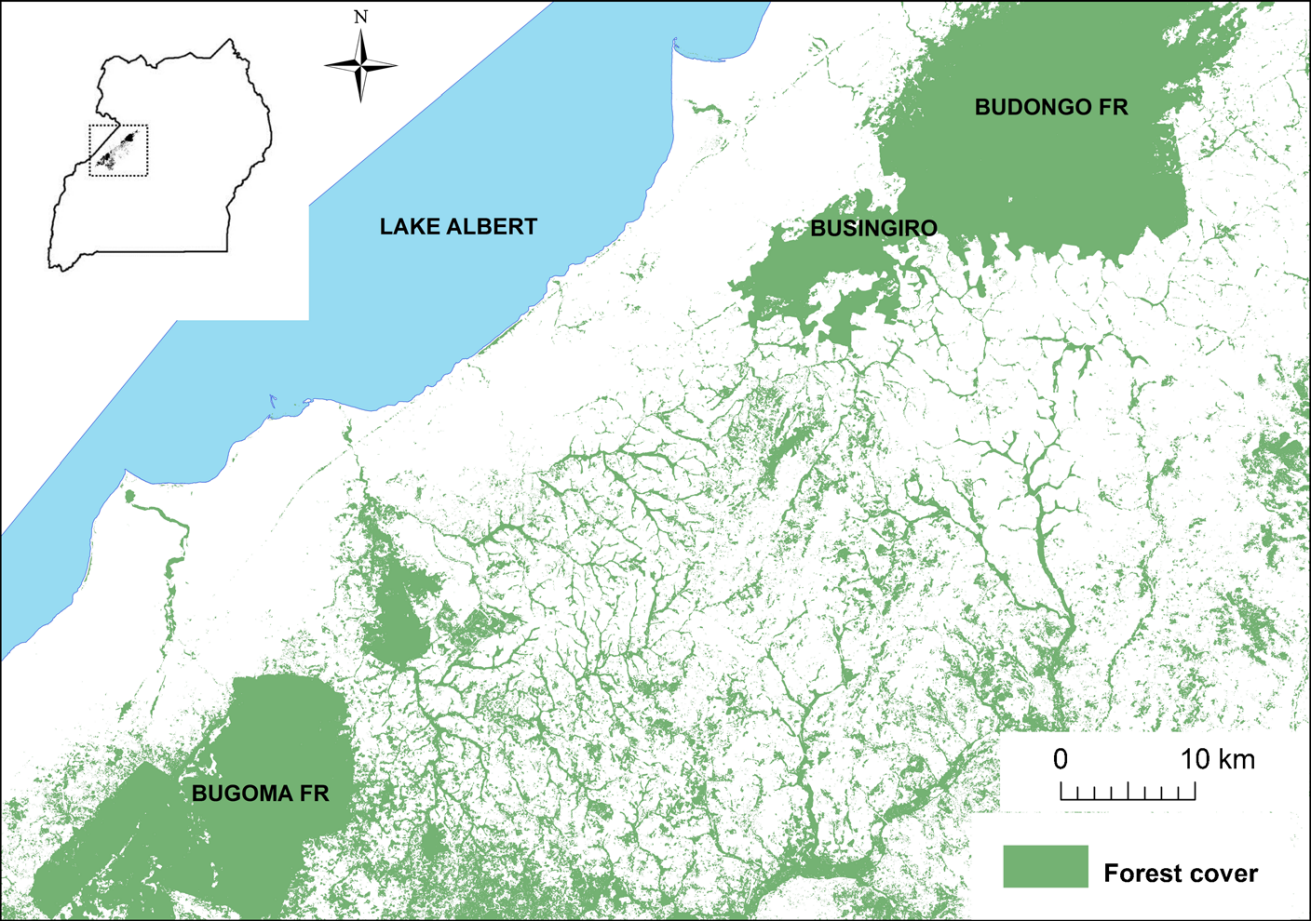
Map of the study area in Uganda. The inset map displays the landscape’s location within Uganda. *Green* indicates forest cover during the study period (Hansen et al. 2013).

We collected data in Hoima and Masindi Districts, Uganda in the corridor area between the Budongo and Bugoma Forests (1°37′–1°68′N and 31°1′–31°6′E; Fig. 1). Both forests are medium-altitude, moist semideciduous forests measuring slightly over 400 km^2^ (Eggeling 1947; Langdale-Brown *et al*. 1964). The region between these forests, which measures roughly 40 km long by 30 km wide, is a mosaic of villages, agricultural land, grasslands, and riparian forest fragments. In the past several decades, human populations have increased substantially in this region, leading to the widespread conversion of unprotected riparian forests for subsistence and commercial agriculture (FAO 2010; Mwavu and Witkowski 2008; Twongyirwe *et al*. 2015). Chimpanzees are broadly distributed in the forested habitat of this region (McCarthy *et al*. 2015; McLennan 2008) and are mostly unhabituated to researcher presence, though they have frequent interactions with their human neighbors (McLennan 2008; McLennan and Hill 2012; McLennan *et al*. 2012).

#### Data Collection

We collected data on chimpanzee nests and feeding traces opportunistically throughout the study area from October 2012 through September 2013 as part of a broader study examining chimpanzee distribution patterns throughout this landscape. We used Landsat satellite imagery to identify the forest fragments located within the boundaries of a given village, and searched permitted and accessible forest fragments within the boundaries of that village. We divided the study area into a grid of 1 km by 1 km cells and recorded when we searched any part of a cell (McCarthy *et al*. 2015). We determined chimpanzee presence based on prior survey evidence (McLennan 2008) and via informal discussion with local inhabitants. Strictly systematic survey methods were impractical in this human-dominated habitat comprising mainly villages and privately owned farms. Instead, we focused search effort in forest fragments on villages within which the privately owned forests were located. In accordance with local customs, before searching a forest fragment we gained permission from the chairperson of the village in which the forest fragment was located, and from individuals identifying themselves as landowners of the forest fragment.

When we encountered nests, we recorded the spatial location of each nest using a Garmin GPSMap® 60CSx. We classified each nest as fresh (all leaves green), recent (some green and some brown leaves), or old (only brown, dead leaves) (Tutin and Fernandez 1984). For each nest, we recorded the tree species name. We also measured tree diameter at breast height (DBH), as well as estimating nest height and tree height using a Bresser® laser Range Finder 800. We noted nests that were constructed using branches from multiple species (composite nests), including the number of plants used for construction and the species of each plant. For ground nests, we recorded whether the nest appeared to be a night nest (well constructed with a thick cushion of support and circular construction) or a day nest (poorly constructed with thin cushioning and noncircular construction) (Brownlow *et al*. 2001; Koops *et al*. 2007; Last and Muh 2013; Stanford and O’Malley 2008). We also recorded the general habitat type in which each nest was found. In addition, we recorded instances in which nests displayed signs of reuse, such as when a previously recorded nest showed signs of fresh use, or when a nest was encountered with fresh leaves woven over a bed of decayed leaves.

We also recorded any feeding traces encountered throughout the study area grid. For each feeding trace, we recorded the spatial location and the species of vegetation consumed. Humans as well as baboons (*Papio anubis*) can leave behind cultivar feeding traces that superficially resemble those produced by chimpanzees. Baboons have mostly been eradicated from the study area, however. In addition, chimpanzee feeding traces were typically easy to identify because they were accompanied by other identifying signs such as chimpanzee knuckle prints, nests, discarded wadges (boluses of chewed vegetation), or feces. For instances in which the consumer of a food item was uncertain, we did not record the feeding trace.

#### Chimpanzee Community Affiliations and Minimum Home Ranges

Based on previously published genetic evidence (McCarthy *et al*. 2015), we associated nesting and feeding patterns with the putative chimpanzee communities in which those behaviors occurred. We constructed 100 % minimum convex polygons (MCPs) for published chimpanzee genotypes found in association using the Minimum Convex Polygon Plugin for QGIS version 2.4.0 (Quantum GIS 2014). We used these MCPs to represent minimum home ranges for each putative chimpanzee community. We then mapped nesting and feeding evidence using QGIS to determine the distribution of nesting and feeding traces associated with each community’s minimum home range. Further details regarding these chimpanzee genotypes and their distributions are available in McCarthy *et al*. (2015).

#### Data Analysis

We analyzed the frequency of nesting across nest ages, habitat types, and tree species. We also examined the distribution of nest tree species for fresh nests only, however, because of the likely variability in nest decay rates across species. We calculated mean nest height as well as the frequency of nest reuse, composite nests, and ground nests. For plantations containing eucalyptus nests, we calculated plantation area using satellite imagery in QGIS version 2.4.0 (Quantum 2014) as well as total forested area within the MCPs of putative chimpanzee communities containing eucalyptus nests (Hansen *et al*. 2013). We also calculated the straight line distance between these eucalyptus nests and the nearest patch of natural forest using spatial measuring tools in QGIS version 2.4.0 (Quantum 2014). We calculated the mean distance between these eucalyptus plantations and the nearest natural forest.

## Ethical Note

We carried out data collection in accordance with the legal requirements of Uganda, and with the permission of the Uganda National Council for Science and Technology, the Uganda Wildlife Authority, and the National Forestry Authority of Uganda. Additional permissions were granted by local landowners where applicable, as described previously. Because data collection was entirely noninvasive and required no contact with the chimpanzees, ethical consent was not necessary for this project.

## Results

### Chimpanzee Nests

**Fig. 2:**
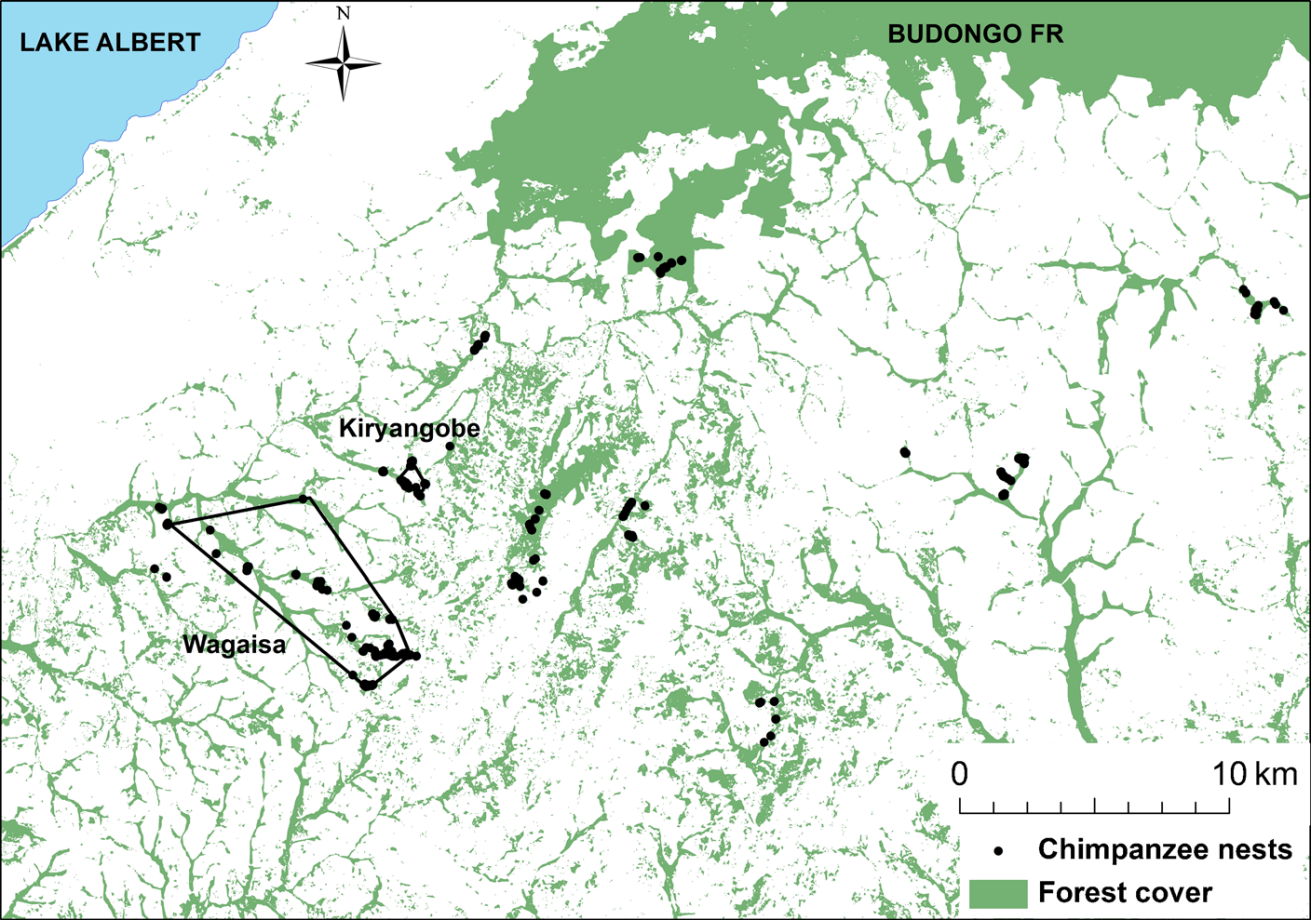
Chimpanzee nest locations throughout the study area, western Uganda, from October 2012 to September 2013. Individual chimpanzee nests are indicated as black circles. Not all nests are visible because of map scaling. MCPs for Wagaisa and Kiryangobe are indicated by polygons. *Green* indicates forest cover during the study period (Hansen et al. 2013).

We recorded a total of 881 nests throughout the study area (Fig. 2). Of these, 489 (56 %) were fresh nests, 269 (31 %) were recent nests, 105 (12 %) and were old nests. For an additional 18 nests (2 %), ages could not be estimated. Among the seven recorded habitat types, nests were most commonly encountered in riparian forests (270 nests; 31 % of total nests; Table I).

**Table I:** Habitat types in which chimpanzee nests were encountered in Hoima and Masindi Districts, Uganda from October 2012 to September 2013

Habitat type	No. of nests	Proportion of total nests
Plantation	198	0.22
Permanently inundated swamp forest	202	0.22
Med. alt. rain forest	174	0.19
Forest edge	6	0.01
Farmland	5	0.01
Riparian forest	290	0.32
Woodland	26	0.03

Mean nest height was 10.9 m (SD = 5.5 m; range: 0–33 m; Table II). We recorded a total of 19 ground nests (2 % overall), including both those classified as day nests and as night nests. Of these, we classified 11 (1 %) as night nests. Fifty-nine nests (7 %) were composite nests constructed with branches from more than one plant. In addition, 35 nests (4 %) displayed evidence of reuse.

**Table II:** Chimpanzee nest characteristics across various study sites

Study location	General habitat type	Nest height (m)^a^	SD	Range	Source
Fongoli, Senegal	Savanna woodland	8.3	4.1		Pruetz et al. (2008)
Kalinzu Forest, Uganda	Medium-altitude evergreen forest	9.5			Furuichi and Hashimoto (2000)
Kahuzi-Biega, DRC	Montane forest	9.8	3.2		Basabose and Yamagiwa (2002)
Equatorial Guinea	Primary forest	10 (median)			Baldwin et al. (1981)
Budongo-Bugoma Corridor, Uganda	Agricultural-riparian mosaic	10.9	5.5	0–33	This study
Semliki, Uganda		11	5.8	1.5–47.8	Hunt and McGrew (2002)
Assirik, Senegal	Savanna woodland	11.3	5.2	2–40	Baldwin (1979)
Seringbara, Guinea	Medium altitude evergreen forest	11.3	6.3		Koops et al. (2012)
Lope, Gabon		11.7		2–45	Wrogemann (1992)
Sapo, Liberia	Lowland mixed forest	12 (median)			Anderson et al. (1983)
Budongo, Uganda	Medium-altitude semideciduous forest	12.1			Brownlow et al. (2001)
Kibira, Burundi	Tropical highland forest	12.1	5.8	3–33.2	Hakizimana et al. (2015)
Issa, Tanzania	Savanna woodland	12.2	4.2	1.2–33.0	Hernandez-Aguilar et al. (2013)
Petit Loango, Gabon		12.5			Furuichi et al. (1997)
Ugalla, Tanzania	Savanna woodland	13.4	5.1	3–30	Ogawa et al. (2007)
Ishasha, Democratic Republic of Congo	Gallery forest	13.5	6.1		Sept (1992)
Assirik, Senegal	Savanna woodland	13.6			Pruetz et al. (2008)
Ntakata/Kakungu, Tanzania	Savanna woodland	13.9		4–30	Ogawa et al. (2006)
Lagoas de Cufada National Park, Guinea-Bissau	Mosaic dense and open canopy forest, savanna-woodland	14.6	0.01		Carvalho et al. (2015)
Bwindi, Uganda	Montane evergreen forest	16.1	6.2		Stanford and O’Malley (2008)
Lagoas de Cufada National Park, Guinea-Bissau	Mosaic dense and open canopy forest, savanna-woodland	16.1	5.2		Sousa et al. (2014)
Goualougo Triangle, Republic of Congo	Lowland rainforest	17.3	7.4		Sanz et al. (2007)
Cantanhez National Park, Guinea-Bissau	Mosaic	19.7	2.8	5–30	Sousa et al. (2011)

^a^Nest heights are mean values unless otherwise reported.

We recorded nests in a total of 49 plant species. When we considered nests irrespective of age, *Eucalyptus grandis* was the most common nest tree species, followed by *Macaranga schweinfurthii* and *Pseudospondias microcarpa* (Table III). The same three species were the most commonly encountered for fresh nests, though nests of *Macaranga schweinfurthii* were encountered slightly more than *Eucalyptus* overall. In addition to eucalyptus, the chimpanzees used six other introduced species for nesting: cocoa (*Theobroma cacao*), jackfruit (*Artocarpus heterophyllus*), mango (*Mangifera indica*), Caribbean pine, (*Pinus caribaea*), guava (*Psidium guajava*), and sugar cane (*Saccharum officinarum*). Nests in introduced species composed 22 % of total nests overall (Table III).

**Table III:** Chimpanzee nest tree species and their relative frequency of use in both fresh nests and total nests, Hoima and Masindi Districts, Uganda, October 2012 to September 2013

Tree species	All nests, regardless of age		Fresh nests only	
	No. of nests	Prop. of nests	No. of nests	Prop. of nests
***Eucalyptus grandis***	**180**	**0.204**	**66**	**0.135**
*Macaranga schweinfurthii*	124	0.141	77	0.157
*Pseudospondias microcarpa*	75	0.085	50	0.102
*Tabernaemontana pachysiphon*	34	0.039	18	0.037
*Oxyanthus speciosus*	29	0.033	8	0.016
*Mitragyna stipulosa*	28	0.032	20	0.041
*Funtumia africana*	19	0.022	17	0.035
*Phoenix reclinata*	17	0.019	9	0.018
*Pycnanthus angolensis*	15	0.017	9	0.018
*Lovoa trichilioides*	12	0.014	11	0.022
*Maerua spp.*	12	0.014	4	0.008
*Piptadeniastrum africanum*	12	0.014	12	0.025
*Myrianthus arboreus*	11	0.012	7	0.014
*Rinorea spp.*	11	0.012	4	0.008
*Sapium ellipticum*	11	0.012	2	0.004
*Morus spp.*	11	0.012	5	0.010
*Khaya anthotheca*	10	0.011	9	0.018
***Theobroma cacao***	**10**	**0.011**	**7**	**0.014**
*Albizia zygia*	7	0.008	6	0.012
*Trilepisium madagascariensis*	6	0.007	4	0.008
*Alchornea laxiflora*	6	0.007	4	0.008
*Ficus spp.*	6	0.007	6	0.012
*Combretum spp.*	5	0.006	0	0.000
*Acacia hockii*	4	0.005	4	0.008
*Ficus vallis-choudae*	4	0.005	4	0.008
*Parkia filicoidea*	4	0.005	3	0.006
*Antiaris toxicaria*	3	0.003	0	0.000
***Artocarpus heterophyllus***	**3**	**0.003**	**1**	**0.002**
*Cordia millenii*	3	0.003	2	0.004
*Cynometra alexandrii*	3	0.003	1	0.002
*Ficus natalensis*	3	0.003	3	0.006
*Ficus ovata*	3	0.003	0	0.000
*Maesopsis eminii*	3	0.003	3	0.006
*Trichilia dregeana*	2	0.002	0	0.000
*Albizia coriaria*	2	0.002	2	0.004
*Aningeria spp.*	2	0.002	0	0.000
*Entandrophragma spp.*	2	0.002	1	0.002
*Marantochloa leucantha*	2	0.002	1	0.002
*Markhamia lutea*	2	0.002	0	0.000
*Sterculia dawei*	2	0.002	2	0.004
*Vernonia amagdalena*	2	0.002	1	0.002
*Caloncoba schweinfurthii*	1	0.001	1	0.002
*Harungana madagascariensis*	1	0.001	0	0.000
***Mangifera indica***	**1**	**0.001**	**0**	**0.000**
***Pinus caribaea***	**1**	**0.001**	**0**	**0.000**
***Psidium guajava***	**1**	**0.001**	**0**	**0.000**
*Teclea nobilis*	1	0.001	1	0.002
***Saccharum officinarum***	**0**	**0.000**	**0**	**0.000**
Multiple species	60	0.068	34	0.070
*Climbers*	18	0.020	7	0.014
Unknown	97	0.110	63	0.129
Total	881		489	

Bold font indicates introduced species

Owing to the high overall prevalence of eucalyptus nests, we further examined characteristics of nests in this species. We encountered eucalyptus nests in 11 small eucalyptus plantations in the study region. These plantations containing chimpanzee nests were associated with the MCPs of two putative chimpanzee communities (Fig. 2), and covered a total area of 12.3 ha. In contrast, total forest cover for the minimum home ranges of these chimpanzee communities measured 643 ha (Hansen *et al*. 2013). Therefore, fresh eucalyptus nests accounted for 20 % of nests overall and 14 % of fresh nests while eucalyptus trees accounted for *ca.* 2 % of forest cover in the minimum home ranges of these chimpanzee communities. Four additional eucalyptus plantations located near or within known areas of chimpanzee habitat use failed to yield chimpanzee nests during the study period.

We further investigated whether eucalyptus nesting may have been associated with a particularly low availability of suitable nesting trees in the vicinity. The mean distance between eucalyptus plantations used for nesting and the nearest patch of natural forest was 48.2 m (*N* = 11; range: 0–240 m). It was beyond the scope of this study to examine nest tree availability systematically throughout the corridor landscape.

### Eucalyptus Bark Feeding

**Fig. 3:**
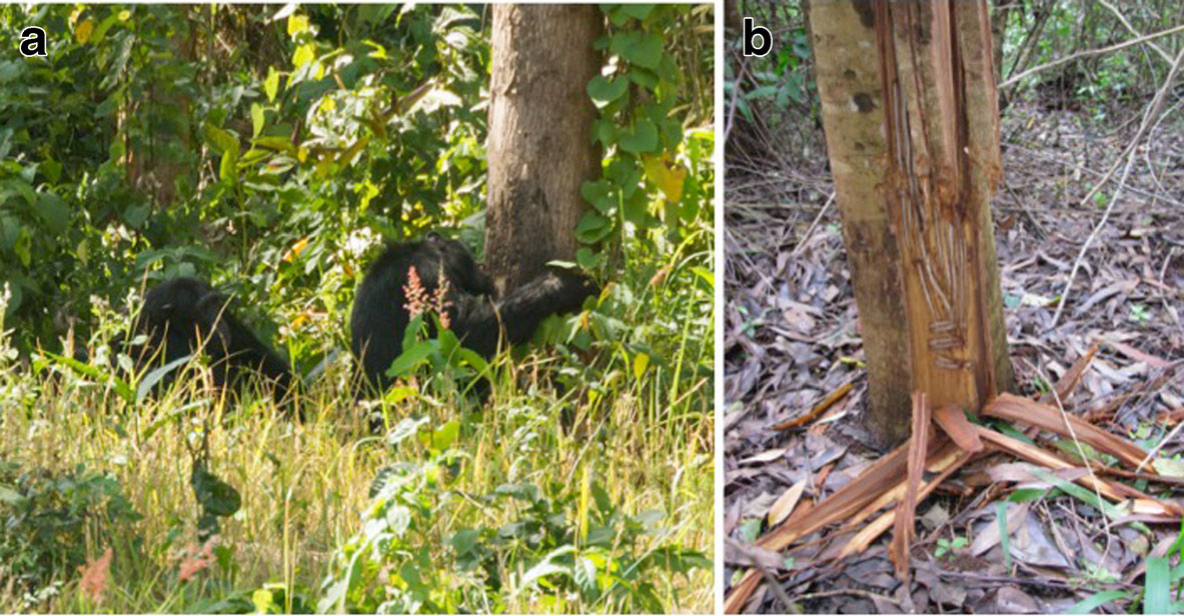
Chimpanzee eucalyptus bark feeding signs and direct observation, Hoima District, Uganda. (**a**) Direct observation of eucalyptus bark feeding by a mixed chimpanzee party, January 2013. (**b**) The base of a eucalyptus tree with evidence of chimpanzee bark stripping.

**Fig. 4:**
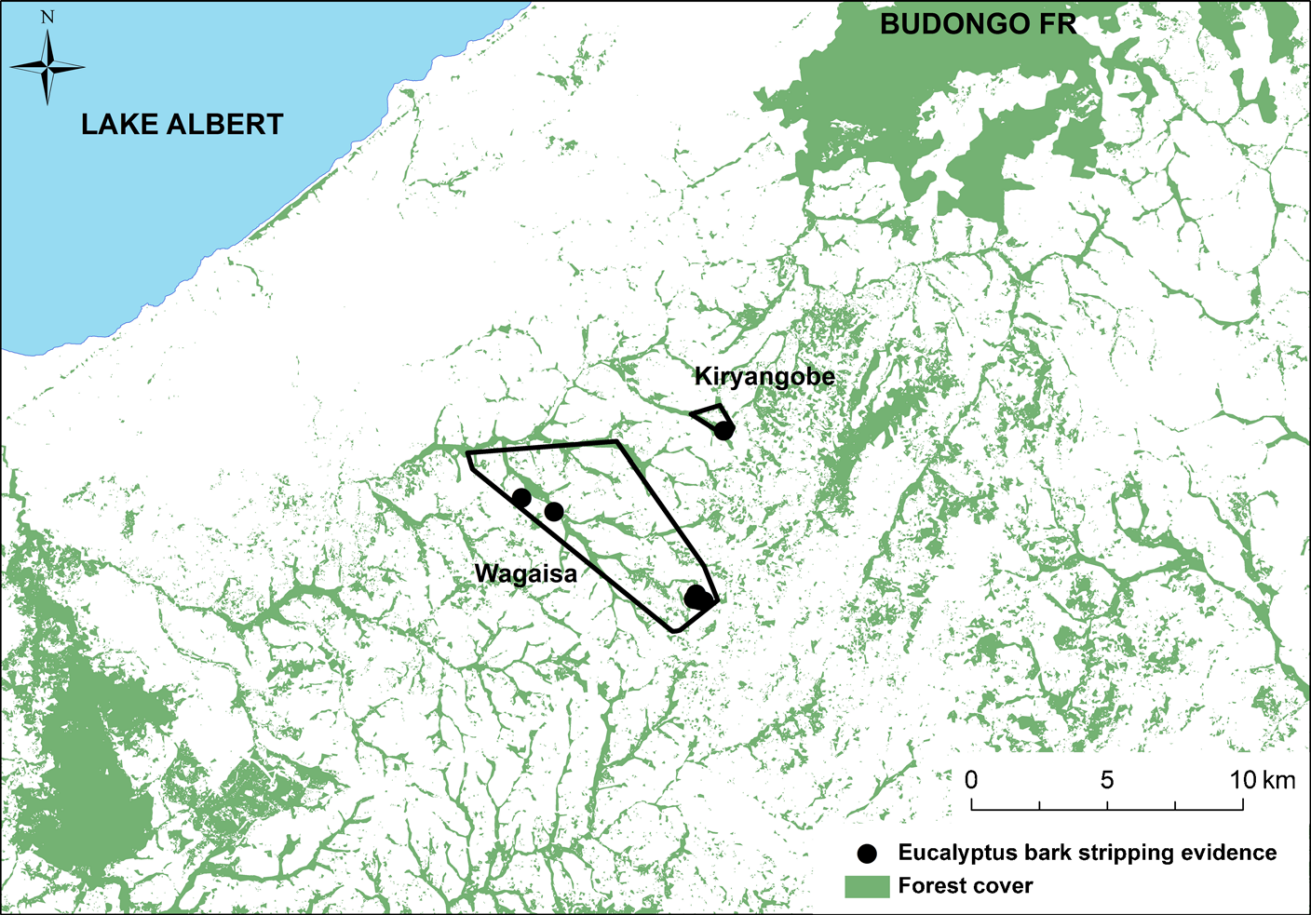
Locations associated with eucalyptus bark feeding evidence during the study period, October 2012 to September 2013. Black circles indicate the locations of chimpanzee bark feeding evidence or direct observations. MCPs for Wagaisa and Kiryangobe are indicated by polygons. *Green* indicates forest cover during the study period (Hansen et al. 2013).

In addition to extensive evidence for the use of introduced species such as eucalyptus for nesting, we also found evidence that chimpanzees consumed eucalyptus bark (Fig. 3). We encountered stripped eucalyptus bark and bark wadges in five eucalyptus plantations in the study area. We found damage only to the bark of eucalyptus trees; we found no evidence of damage to eucalyptus leaves or flowers, and only superficial damage to phloem (incidental to bark stripping; Fig. 3). These plantations fell within the MCPs of the same two putative chimpanzee communities that nested in eucalyptus (Fig. 4). Over the 12-mo study period, we recorded fresh bark stripping evidence across five months (October, January, February, March, and July), during both rainy and dry seasons. We also observed this behavior directly on one occasion (Fig. 3).

## Discussion

In accordance with our hypothesis, chimpanzees in a human-dominated habitat displayed a variety of nesting strategies indicating behavioral responses to anthropogenic disturbance. We predicted that nests would occur at low heights or on the ground in this habitat. Mean nest height was approximately 11 m, which was on the lower end of the range of mean observed nest heights across numerous study sites (Table II). This may indicate that fewer tall trees are available, or that chimpanzees sometimes nest at lower heights to avoid detection by their human neighbors, since low nests in swamps and riparian forests are often difficult to see from outside the forest while higher nests are more easily visible from a distance. Alternatively, chimpanzees may prefer nesting at lower heights and may do so more frequently in dense riparian and swamp forests because nest height is less restricted in this habitat type that humans typically avoid entering.

A small percentage (2 %) of overall nests were night ground nests. This percentage falls within the broad range (0.05–35.4 %) of ground nest rates reported at other sites (reviewed in Tagg *et al*. 2013). Night ground nesting by chimpanzees in southeast Cameroon was associated with human influence and may help chimpanzees avoid human detection in swamps (Tagg *et al*. 2013). Ground nests in this study area may serve a similar function. Predator pressure may play a key role in the absence of ground nesting at some sites (Pruetz *et al*. 2008). In this study area, there are no potential predators present aside from humans, which may also permit occasional ground nesting. Future studies may help clarify factors underlying ground nesting behavior in human-disturbed habitats, including the role of human population density in ground nesting variation.

We also predicted that chimpanzees would nest in introduced species, reuse nests, and construct nests from multiple trees. We observed a small but substantial proportion of reused and composite nests. Nest reuse has sometimes been associated with low nest tree availability or a scarcity of trees with fresh leaves for nesting (Fruth and Hohmann 1996; Wrangham 1992), which may explain its occurrence in this habitat with ongoing deforestation. Composite nests may similarly be used due to the scarcity of suitable nest trees, though the use of such nests is not well documented at other study sites. Nest reuse has also been reported to occur more frequently among chimpanzees with snare injuries in the nearby Budongo Forest (Plumptre and Reynolds 1997). Some chimpanzees in this study area also have missing limbs and other injuries from snares and man-traps and may similarly be more likely to reuse nests for this reason (McLennan *et al*. 2012; Reynolds 2005). Collectively, these findings indicate that chimpanzees are capable of flexibly modifying their nesting strategies in a habitat with shrinking natural forests. These strategies may be driven by necessity, by shifting preferences, or by a combination of factors.

Perhaps most notably, chimpanzees often nested in introduced species. Eucalyptus trees in particular were often used for nest construction, with eucalyptus ranking among the most commonly observed nest tree species. There are several nonmutually exclusive explanations for the common use of eucalyptus for nesting in this region. First, eucalyptus may be used frequently because of its high availability relative to other tree species in the habitat. It was beyond the scope of this study to examine the availability and distribution of nest tree species systematically. Nonetheless, satellite imagery suggested that eucalyptus is overrepresented as a nest tree species given its availability in the habitat. In addition, eucalyptus plantations with nests were typically located in relatively close proximity to natural forest patches; *ca.* 50 m away on average. This indicates that a mere lack of nearby natural forest cannot account for the prevalence of eucalyptus nesting.

Instead, a second potential reason for the high frequency of eucalyptus nesting is that eucalyptus plantations provide easy access to fragments of natural forest that contain important natural food resources. Similarly, cultivars such as jackfruit were sometimes found in close proximity to eucalyptus plantations, which may indicate that eucalyptus acted as a convenient nest species for cultivar feeding. We found widespread evidence that chimpanzees in this habitat consumed cultivars such as jackfruit during the study period, which supports this potential role for eucalyptus nesting (M. McCarthy, *unpubl. data*). Other researchers have similarly suggested that nesting preferences may be driven partially by the close proximity of valuable fruit trees (Brownlow *et al*. 2001; Goodall 1962; Stanford and O’Malley 2008).

Third, eucalyptus may also be used frequently for nesting due to comfort. Pliable branches and small leaves have been identified as potentially important components for nest comfort (Goodall 1962; Hernandez-Aguilar 2009; van Casteren *et al*. 2012). Eucalyptus trees have small leaves, which may make them more comfortable for nesting than some other available tree species. However, the second and third most common nest tree species, *Macaranga schweinfurthii* and *Pseudospondias microcarpa*, typically produce rather large leaves, which may indicate that leaf size does not generally play an important role in nest tree choice. Eucalyptus branches are relatively thin and therefore are likely to be highly pliable, which may suggest eucalyptus makes for comfortable nests, though the physical properties of the branches have not been tested.

Lastly, eucalyptus may be used for nesting because it has antivector properties, which have also been proposed as an important factor influencing chimpanzee nest choice (Koops *et al*. 2012; Samson *et al*. 2013; Stewart 2011). Although the antivector properties of eucalyptus are widely recognized (Ebrahimi *et al*. 2013), the potential for eucalyptus nests to repel insects more effectively than nests constructed in other available tree species needs to be tested.

In addition to nesting in eucalyptus trees, chimpanzees also stripped and wadged the bark of eucalyptus trees in this region. We found fresh eucalyptus bark feeding traces and as well as directly observing this behavior. Numerous primate taxa incorporate bark into their diets (Nishida 1976), and eucalyptus bark feeding in particular has previously been observed in colobus monkeys, mountain gorillas, and Bornean orangutans (Meijaard *et al*. 2010; Rode *et al*. 2003; Rothman *et al*. 2006; Wasserman *et al*. 2012). This study adds chimpanzees to the species of anthropoid primates known to consume eucalyptus bark.

There are several reasons why chimpanzees may consume eucalyptus bark in this habitat. First, eucalyptus may serve as a fallback food. Nishida (1976) reported that tree bark of certain natural tree species served as a fallback food for chimpanzees in the Mahale Mountains, Tanzania during periods of low resource availability. In this study, we did not measure resource availability throughout the habitat and could not test this hypothesis rigorously. However, given the fact that eucalyptus bark consumption occurred at various times throughout the year, in both rainy and dry seasons, and given the relatively low availability of this resource, this explanation seems unlikely. Additionally, we found no evidence for bark feeding in other tree species, indicating that eucalyptus was likely not part of a broader pattern of bark consumption during periods of low resource availability.

Second, eucalyptus may serve a medicinal purpose. Eucalyptus leaves are used by people in Uganda and elsewhere as a treatment for cough (Namukobe *et al*. 2011). Although chimpanzees use numerous plant species for medicinal purposes (Huffman 2001; Masi *et al*. 2012), eucalyptus has not been among those recorded species to date, and its potential use for medicinal purposes in chimpanzees has not been evaluated.

A third possibility is that chimpanzees consume eucalyptus bark because of its high mineral content. In particular, eucalyptus bark consumption in mountain gorillas and colobus monkeys has been attributed to its particularly high sodium content (Nkurunungi 2004; Rode *et al*. 2003; Rothman *et al*. 2006; Wasserman *et al*. 2012). Eucalyptus bark also contains secondary compounds such as phytoestrogens, however, which may act as endocrine disruptors or potentially have other detrimental effects on consumers (Wasserman *et al*. 2012, 2013). The effects of eucalyptus secondary compounds on chimpanzees are currently not known.

Chimpanzees in this fragmented forest landscape use introduced species, in particular eucalyptus, for both nesting and feeding. Eucalyptus may be used due to its relative availability as a nesting and feeding resource, or for a variety of other nonmutually exclusive reasons. Interestingly, these behaviors were observed in just two putative chimpanzee communities during the study period despite the presence of eucalyptus plantations in other areas of chimpanzee habitat use. More recently, eucalyptus nesting and bark consumption has also been observed at a third site in the survey region, Bulindi (M. McLennan, *pers. comm*.). This variation may reflect differences in search effort or a relatively higher availability of eucalyptus in communities that use it. It could also potentially reflect local community-level variation in behavioral traditions for chimpanzee communities introduced to novel resources. Further studies may help clarify both the role of introduced species and the potential of these species to support great apes in degraded habitats. Given the increasing prevalence of such habitats throughout the geographic ranges of great apes, introduced species are likely to become increasingly relied on in remaining populations. Rather than representing rare and novel behavioral variants, the use of such species may become the norm for some surviving great ape populations in degraded habitats of the Anthropocene. The potential for such behavioral flexibility to support the long-term persistence of great apes in degraded habitats remains highly uncertain, however.
